# PRC2-Mediated H3K27me3 Contributes to Transcriptional Regulation of FIT-Dependent Iron Deficiency Response

**DOI:** 10.3389/fpls.2019.00627

**Published:** 2019-05-16

**Authors:** Emily Y. Park, Kaitlyn M. Tsuyuki, Fengling Hu, Joohyun Lee, Jeeyon Jeong

**Affiliations:** ^1^Program in Biochemistry and Biophysics, Amherst College, Amherst, MA, United States; ^2^Department of Biology, Amherst College, Amherst, MA, United States

**Keywords:** iron, FIT, H3K27me3, PRC2, transcription, Arabidopsis

## Abstract

Iron is an essential micronutrient for nearly all organisms, but excessive iron can lead to the formation of cytotoxic reactive oxygen species. Therefore, iron acquisition and homeostasis must be tightly regulated. Plants have evolved complex mechanisms to optimize their use of iron, which is one of the most limiting nutrients in the soil. In particular, transcriptional regulation is vital for regulating iron in plants, and much work has revealed the role of transcription factors on this front. Our study adds novel insights to the transcriptional regulation of iron homeostasis in plants by showing that chromatin remodeling via histone 3 lysine 27 trimethylation (H3K27me3) modulates the expression of FIT-dependent genes under iron deficiency. We provide evidence that FIT-dependent iron acquisition genes, *IRT1* and *FRO2*, as well as *FIT* itself are direct targets of PRC2-mediated H3K27me3. In the *clf* mutant, which lacks the predominant H3K27 tri-methyltransferase, induction of *FIT*, *FRO2*, *IRT1*, and other FIT-regulated genes in roots is significantly higher under iron deficient conditions than in wild type. Furthermore, we observe that *clf* mutants are more tolerant to iron deficiency than wild type, indicating that gene expression levels appear to be limiting the plants ability to access iron. We propose that H3K27me3 attenuates the induction of FIT-target genes under iron deficiency and hypothesize that this may serve as a mechanism to restrict the maximum level of induction of iron acquisition genes to prevent iron overload.

## Introduction

Much of the micronutrients in the earth’s soil is inaccessible to plants, which negatively affects crop yield and quality (Marschner, 2012). In particular, iron is abundant in the earth’s crust but is one of the most limiting nutrients for plant growth because it exists as insoluble ferric oxides under aerobic and neutral or alkaline conditions (Colombo et al., 2014). Plants have a unique need for iron as photosynthetic organisms; however, they are exposed to a higher risk of iron-induced toxicity. Hence, plants have evolved a host of delicate mechanisms to tightly control iron acquisition and homeostasis. To improve agriculture and human health, it is crucial to understand the molecular mechanisms behind iron acquisition and regulation in plants.

Dicots, including *Arabidopsis thaliana*, use Strategy I, which implements a reduction-based mechanism for acquiring iron (Jeong et al., 2017). Under iron-deficient conditions, *A. thaliana* extrudes protons through the P-type H^+^-ATPases, such as AHA2, and acidifies the rhizosphere to help solubilize Fe^3+^-chelates (Santi and Schmidt, 2009). PDR9, an ABC transporter, facilitates the export of coumarin-family phenolics into the surrounding rhizosphere and contributes to the formation of an accessible pool of iron in the apoplast (Fourcroy et al., 2014; Clemens and Weber, 2016). FERRIC REDUCTASE OXIDASE 2 (FRO2) reduces ferric iron-chelates to ferrous iron (Robinson et al., 1999), which is then transported into the root epidermal cell by Iron-Regulated Transporter 1 (IRT1) (Connolly et al., 2002; Varotto et al., 2002; Vert et al., 2002).

The iron acquisition process is largely controlled by a sophisticated transcriptional regulatory network. FER-LIKE IRON DEFICIENCY-INDUCED TRANSCRIPTION FACTOR (FIT), the master regulator of the iron deficiency response, is essential for the high-level induction of Strategy I iron acquisition genes and *FIT* loss-of-function mutants are lethal under iron deficient conditions (Colangelo and Guerinot, 2004; Jakoby et al., 2004; Yuan et al., 2005). FIT is a basic-helix-loop-helix (bHLH) transcription factor that dimerizes with bHLH subgroup Ib transcription factors, bHLH38, bHLH39, bHLH100, or bHLH101 to positively regulate its target genes (Yuan et al., 2008; Wang et al., 2013). However, rather than inducing FIT target genes, bHLH100 and bHLH101 may regulate genes involved in distributing iron in tissues and organelles via a FIT-independent pathway (Sivitz et al., 2012). Meanwhile, FIT also interacts with transcription factors of other regulatory networks involving hormones, such as jasmonic acid, ethylene, or gibberellin, as well as bHLH transcription factors of bHLH IIIe and IVa subgroups that control FIT at the transcriptional and posttranslational levels (Lingam et al., 2011; Wild et al., 2016; Naranjo-Arcos et al., 2017; Cui et al., 2018; Tanabe et al., 2018).

Evidence suggests that there are mechanisms upstream of FIT that control iron acquisition. FIT itself is iron-regulated at the transcriptional and post-transcriptional levels. *FIT* is induced by iron deficiency (Colangelo and Guerinot, 2004), but in plants that constitutively express *FIT*, FIT subjected to rapid turnover by proteasomal degradation at the early onset of the iron deficiency response (Sivitz et al., 2011). Recently, an iron-regulated calcium dependent protein kinase, CBL-INTERACTING PROTEIN KINASE (CIPK) 11, was identified as a positive regulator of FIT (Gratz et al., 2019). Meanwhile, IRON MAN (IMA) peptides, also referred as FE-UPTAKE-INDUCING PEPTIDEs (FEPs), were reported as an upstream regulator of the iron uptake pathway (Grillet et al., 2018; Hirayama et al., 2018). Overexpression of the IMA motif was sufficient to trigger the iron deficiency response and lead to accumulation of iron (Grillet et al., 2018), but IMA3/FEP1 was proposed to activate bHLH38/39 via a mechanism independent of FIT, with the expression of FEP1 also being independent of FIT (Hirayama et al., 2018).

In addition to FIT, POPEYE (PYE) is another bHLH transcription factor that plays a major role under iron deficiency (Long et al., 2010). PYE represses genes, such as *NICOTIANMINE SYNTHASE 4* (*NAS4*) and *FERRIC REDUCTASE OXIDASE 3* (*FRO3*), which are involved in root iron mobilization and shoot translocation. NAS4 synthesizes nicotianamine, which chelates iron into a mobile form for vascular translocation (Bauer et al., 2004), and FRO3 is a mitochondrial ferric chelate reductase expressed in the vasculature (Jeong and Connolly, 2009). The expression of *PYE* is tightly correlated with *bHLH39* and *bHLH101* (Long et al., 2010), which are strongly induced by iron deficiency (Yuan et al., 2008). Because bHLH039 is a FIT binding partner, PYE activity mediates widespread transcriptional regulation under iron deficiency through indirect regulation of FIT or with direct interaction with other PYE targets (Long et al., 2010). Although *PYE* is most highly expressed in the root pericycle, PYE protein is found in the nuclei of all root cells under iron deficiency (Long et al., 2010), which implies that PYE might translocate throughout the root and convene with the FIT network. *PYE* is a direct target of bHLH34, and *FIT* and *bHLH38/39/100/101* were also proposed as direct targets of bHLH34 (Li et al., 2016).

Even though the intricate transcriptional network of the iron deficiency response has been extensively studied (Dinneny et al., 2008; Buckhout et al., 2009; Rodríguez-Celma et al., 2013; Mai et al., 2016; Gao et al., 2019), the effect of chromatin structure on iron homeostasis gene expression is not well-understood. In addition to transcription factors, chromatin structure is a major transcriptional regulator in eukaryotes (Li et al., 2007). The covalent chemical modification of the histone tails that modifies the compression and relaxation of the chromatin directly impacts transcription factor and transcriptional machinery access to promoter regions, and thus subsequent gene expression.

Histone 3 lysine 27 trimethylation (H3K27me3) is a well-established repression mark, controlled by the activity of polycomb-group (PcG) protein complexes that play significant roles in regulating gene expression and multicellular development (Köhler and Hennig, 2010; Pu and Sung, 2015). For example, the homeotic MADS box gene *AGAMOUS* (*AG*) is involved in specifying floral organ identity (Bowman et al., 1989; Yanofsky et al., 1990) and has been shown to be regulated by Polycomb Repressive Complex 2 (PRC2)-mediated H3K27me3 activity (Goodrich et al., 1997; Katz et al., 2004). In Arabidopsis, PRC2 specifically catalyzes the trimethylation of H3K27 (Zhang et al., 2007). Silencing occurs when PRC2 trimethylates K27 on histone 3, and the repression mark spreads along the chromatin, ultimately resulting in its compaction (Margueron and Reinberg, 2011). The CURLY LEAF (CLF) and SWINGER (SWN) subunits are responsible for the PRC2 methyltransferase activity in Arabidopsis. While CLF and SWN are partially redundant, CLF is the predominant H3K27 tri-methyltransferase of PRC2 and loss of CLF function results in drastically reduced H3K27me3 deposition (Chanvivattana et al., 2004; Schubert et al., 2006; Zhang et al., 2007; Wang et al., 2016); thus, *clf* mutants are widely used as PCR2 mutants (Lafos et al., 2011; Lu et al., 2011; Zhou et al., 2018). Nearly 20% of Arabidopsis genes were identified as H3K27me3 targets, which predominantly consisted of transcriptionally repressed genes (Turck et al., 2007; Zhang et al., 2007). In plants, H3K27me3 has been understood as a widely used repressive epigenetic mark in response to not only developmental cues, but also abiotic stressors such as temperature and drought (Probst and Mittelsten Scheid, 2015; Secco et al., 2017).

In this study, we show that post-translational modification of a core histone, histone 3 lysine 27 trimethylation (H3K27me3), modulates the induction of *FIT* and FIT-dependent iron acquisition genes under iron deficiency. Our work reveals that FIT-dependent genes are more pronouncedly induced by iron deficiency in the *clf* mutant, which lacks the major H3K27 tri-methyltransferase, than in wild type. Furthermore, we show that *FIT*, *FRO2*, and *IRT1* are direct targets of H3K27me3 and that the *clf* loss-of-function mutant grows better than wild type in conditions with low levels of iron.

## Materials and Methods

### Plant Materials and Growth Conditions

*Arabidopsis thaliana* ecotype Columbia (Col-0) was used as the wild type. The *clf* mutant line used was *clf-29* (SALK_21003) from the Arabidopsis Biological Resource Center. Arabidopsis seeds were surface-sterilized and plated on B5 media. After stratification, seeds were grown at 22°C under a 16/8 h light/dark cycle in a Conviron A1000 growth chamber. For iron treatments, plants were transferred to iron deficient media (-Fe), Murashige and Skoog (MS) medium with no iron (Caisson MSP33) supplemented with 300 μM ferrozine, or Fe sufficient media (+Fe), MS medium with 100 μM FeNa-EDTA (Caisson MSP34) for 3 days for both gene expression analysis and chromatin immunoprecipitation.

### RNA Extraction

Total RNA was extracted from root and shoot tissues using the Plant RNA Isolation Kit (Agilent) per the manufacturer’s instructions. The concentration and purity of RNA were measured with NanoDrop One (Thermo Fisher Scientific), and the integrity of RNA was assessed by running a bleach gel (Aranda et al., 2012).

### Quantitative RT-PCR

cDNA was synthesized from total RNA using iScript^TM^ Reverse Transcription Supermix (Bio-Rad) following manufacturer’s instructions. cDNA samples were then diluted 1:10 with nuclease-free water and used for RT-qPCR analysis with iTaq^TM^ Universal SYBR© Green Supermix (Bio-Rad) following manufacturer’s instructions using the CFX Connect^TM^ Real-Time System (Bio-Rad). A melt curve analysis was performed at the end of the qPCR protocol. Relative transcript levels of genes of interest was calculated using the ΔCt method (Schmittgen and Livak, 2008). The expression level of *ACTIN2* (*ACT2*) was used as an internal control. Primers were designed using QuantPrime (Arvidsson et al., 2008). Primer sequences are listed in Supplementary Table S1.

### RNA-Sequencing and Data Analysis

Three biological replicates of wild type and *clf* roots grown under iron deficient or sufficient conditions were used for RNA-seq analysis. RNA was extracted as described above. cDNA library construction, 150 bp paired-end sequencing on Illumina NovaSeq 6000 platform, and standard RNA-seq analysis were conducted by Novogene. Gene expression was quantified using the union mode of the HTSeq software and reported as FPKM (Fragments Per Kilobase of transcript sequence per Millions base pairs sequenced). All sequencing reads were aligned to the *Arabidopsis thaliana* reference genome, TAIR10^1^. Readcount histograms of the genes of interest was generated using the Integrative Genomics Viewer (IGV), IGV 2.4 (Robinson et al., 2011; Thorvaldsdóttir et al., 2013). Differential gene expression analysis was conducted using DESeq with a threshold set as padj <0.05 using negative binominal distribution for *p*-value estimation and BH for FDR estimation (Anders and Huber, 2010). The Gene Ontology (GO) term annotations were conducted using the enrichment analysis tool (Mi et al., 2017) available via from the Gene Ontology Consortium (Ashburner et al., 2000; The Gene Ontology Consortium, 2017). Statistical analyses of iron regulated genes were performed using R (The R Foundation, Vienna, Austria). The genes used for the analysis shown in Figure 2 are listed in Supplementary Table S2.

### Chromatin Immunoprecipitation (ChIP) – qPCR

Wild type and *clf* plants were grown as described above and root tissue was harvested. Native proteins were crosslinked with gDNA using GB buffer (0.4 M sucrose, 10 mM Tris pH 8.0, 1 mM EDTA), 100 mM PMSF, and 37% formaldehyde. Plant tissue was ground in liquid nitrogen by mortar and pestle and sonicated in lysis buffer (500 mM HEPES pH 7.5, 150 mM NaCl, 1 mM EDTA, 1% Triton X-100, 0.1% deoxycholate, 0.1% SDS). Crosslinked DNA was precleared with Protein A Agarose/Salmon Sperm DNA beads (Millipore Sigma, 16-157) for 1 h at 4°C with gentle rotation and then incubated with anti-IgG (Millipore Sigma, 12-370), H3 (AbCam 1791), or H3K27me3 antibodies (Millipore Sigma, 07-449) overnight at 4°C with gentle rotation. Crosslinked DNA was washed in a series with lysis buffer, LNDET buffer (0.25 M LiCl, 1% NP-40, 1% deoxycholate, 1 mM EDTA, 10 mM Tris-HCl pH 8.0), and TE buffer before elution with elution buffer (1% SDS, 0.1 M NaHCO_3_). Crosslinking was reversed with Proteinase K overnight at 65°C, and DNA was purified using the Zymo-Spin ChIP Kit per manufacturer’s instructions. The qPCR was conducted as described above, and gene enrichment was normalized to 10% of input (Haring et al., 2007). Primers were designed using NCBI Primer-BLAST (Ye et al., 2012). Primer sequences are listed in Supplementary Table S3.

### Low Iron Growth

Wild type and *clf* seeds were surface-sterilized, plated, and grown under light and temperature conditions as described above after stratification. Plants were grown on media with no added iron and 300 μM ferrozine, low iron (10 μM Fe), or sufficient iron (100 μM Fe) for up to 12 days. Low iron media was prepared by adding sterile 10 μM Fe (III)-NaEDTA to MS medium without iron (Caisson MSP33). Root lengths were measured using ImageJ.

## Results

### Iron Acquisition Genes Are More Highly Induced in Roots of *clf* Than in Wild Type

Multiple large-scale epigenomics studies have mapped H3K27me3 target loci in Arabidopsis (Turck et al., 2007; Zhang et al., 2007; Bouyer et al., 2011; Lafos et al., 2011; Lu et al., 2011; Roudier et al., 2011). After a survey of the genome-wide studies, we noted that loci of several iron deficiency response genes, including the major iron acquisition genes, were among those identified to be associated with H3K27me3 (Table 1). Such findings suggested that iron homeostasis genes are under the control of H3K27me3 and led us to hypothesize that Arabidopsis might implicate chromatin remodeling to regulate the iron deficiency response. To test this hypothesis and understand the role of H3K27me3 in the iron deficiency response, we examined the expression of iron homeostasis genes that are well-established to be induced under iron deficiency in wild type and *clf* mutant roots. CLF is a core PRC2 component in Arabidopsis, which is largely responsible for the methyltransferase activity of PRC2 (Chanvivattana et al., 2004; Köhler and Hennig, 2010), and *clf* mutants are used as PRC2 mutants (Lafos et al., 2011; Lu et al., 2011; Zhou et al., 2018). Although CLF is partially redundant with SWN, we did not conduct our experiments using the *clf swn* double mutant, which exhibits severe developmental phenotypes and develops into a mass of callus-like tissue that does not differentiate into roots and shoots (Chanvivattana et al., 2004). Because our study focuses on root-specific iron acquisition genes, we conducted experiments using a *clf* single mutant, *clf-29*, which has remarkably reduced levels of H3K27me3 and has been widely used in multiple studies (Bouveret et al., 2006; Schönrock et al., 2006; Xu and Shen, 2008; Zhao et al., 2015; de Lucas et al., 2016; Bellegarde et al., 2018). In addition, we verified expression of iron deficiency response genes and low iron growth phenotypes of a second *clf* mutant, *clf-28*, as shown in Supplementary Figures S1, S4.

**Table 1:** Iron deficiency response genes identified as H3K27me3 targets from existing large scale epigenomic datasets.

Gene ID	Gene name	Annotated function
At1g01580	*Ferric Reductase Oxidase 2* (*FRO2*)	Reduces ferric-chelate iron to ferrous iron
At2g28160	*FER-like iron deficiency transcription factor* (*FIT*)	Regulator of the iron deficiency response
At3g08040	*Ferric Reductase Defective 3* (*FRD3*)	Cation transmembrane transport
At3g13610	*F6’H1*	Iron uptake and production of iron uptake-related fluorescent phenolics
At3g50740	*UDP-Glucosyl Transferase 72E1* (*UGT72E1*)	UDP-glucosyl transferase activity, response to toxic substances
At3g53480	*Pleiotropic Drug Resistance 9* (*PDR9*)/*ATP-binding cassette G37* (*ABCG37*)	Secretion of iron uptake-related fluorescent phenolics
At4g19680	*Iron Regulated Transport 2* (*IRT2*)	Ferrous iron ion transporter
At4g19690	*Iron Regulated Transport 1* (*IRT1*)	Ferrous iron ion transporter


If iron deficiency response genes are repressed by H3K27me3, their expression will be higher in the *clf* mutant than in wild type. We predicted that these genes might be induced at a higher level in *clf* than in wild type under iron deficient conditions, but still remain at a low basal level under iron sufficient conditions in *clf*. Previous studies have shown that the removal of H3K27me3 is often not sufficient to induce gene expression (Bouyer et al., 2011), and FIT overexpression studies have shown that FIT is inactive under iron sufficiency (Lingam et al., 2011; Meiser et al., 2011). Thus, it is likely that an upstream regulatory mechanism that is activated by the iron deficiency signal will be necessary to trigger the induction of iron acquisition genes.

**FIGURE 1:**
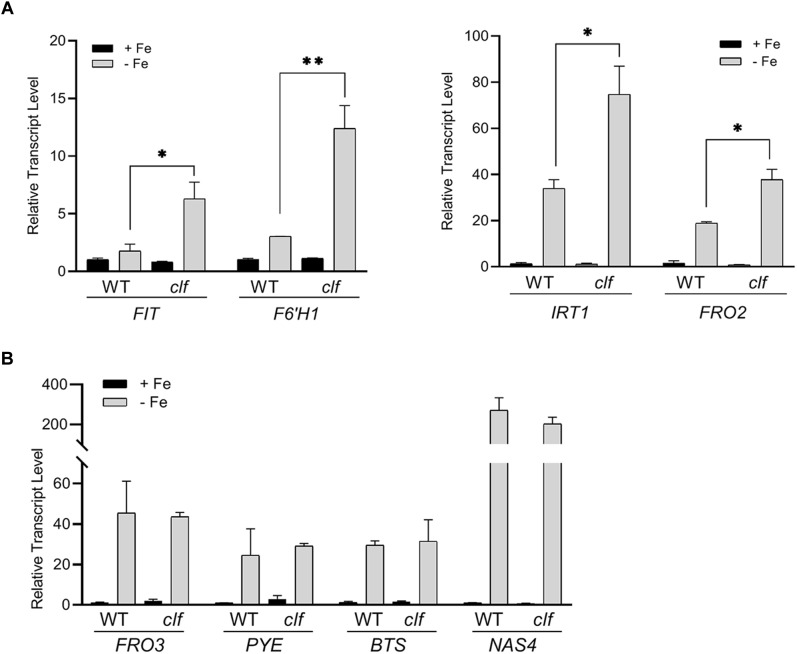
Transcript levels of iron deficiency inducible genes in wild type and *clf* roots. Quantitative RT-PCR was conducted with total RNA extracted from roots of 12-day-old plants grown on B5 without sucrose and then transferred to iron sufficient (+Fe; 100 μM Fe) or iron deficient (–Fe; 300 μM ferrozine) media for 3 days. Transcript levels were normalized to an internal control, *ACT2*, and reported as the expression ratio relative to wild type under iron-sufficient conditions. **(A)** FIT-dependent genes, **(B)** PYE-dependent genes. Mean values of three biological replicates are shown. Error bars represent standard error (*n* = 3).^∗^*p* < 0.05; ^∗∗^*p* < 0.01.

To test if iron deficiency response genes are differentially regulated in the *clf* mutant, we first quantified the expression of FIT-target genes involved in iron acquisition such as *FIT*, *IRT1*, *FRO2*, and *F6’H1*, in *clf* and wild type roots from iron sufficient and deficient conditions. As expected, in wild type, these genes were highly induced under iron deficiency but barely expressed under iron sufficient conditions (Figure 1A). In *clf* roots from iron sufficient conditions, expression of FIT-target genes remained low, consistent with the wild type (Figure 1A). However, the induction of *FIT*, *IRT1*, *FRO2*, and *F6’H1* was significantly higher in iron deficient roots of *clf* than in those of wild type (Figure 1A). The higher induction of FIT target genes under iron deficient conditions was consistently observed in the second *clf* mutant (Supplementary Figure S1). This suggests that H3K27me3 is primarily involved in modulating Strategy I genes under iron deficiency and prevents hyper-induction of these genes.

Next, we examined PYE-dependent genes, *FRO3*, *NAS4*, and *BTS*, which are also known to be induced under iron deficiency (Long et al., 2010). The PYE-dependent genes were highly induced under iron deficient conditions in both wild type and *clf* roots, but their expression levels were comparable between both genotypes (Figure 1B). Our qRT-PCR data suggested that H3K27me3 modulates the induction of FIT-dependent genes in response to iron deficiency, whereas PYE-dependent genes are not likely to be under the control of this repression mark under iron deficient conditions. The results also revealed that H3K27me3 is not the primary factor that represses iron deficiency inducible genes under iron sufficient conditions, as neither the FIT- nor PYE-regulated genes tested were expressed in the *clf* mutant in the presence of iron (Figure 1).

### Transcriptomic Analysis of Genes Differentially Regulated by Iron in Wild Type and *clf* Roots

**FIGURE 2:**
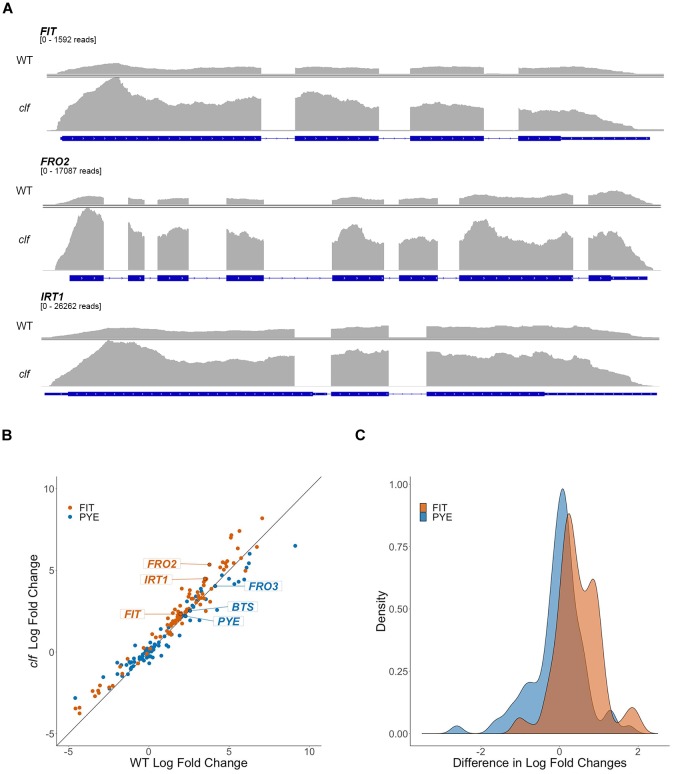
Differential regulation by iron of FIT-dependent iron acquisition genes in *clf* compared to wild type. **(A)** RNA-seq read count histograms of FIT-dependent iron acquisition genes in wild type and *clf* under iron deficiency. Gene diagrams depict introns (line) and exons (boxes) and are aligned with read counts. The read count range, denoted below the gene name, were scaled to the maximum number of reads obtained in *clf*. Read count scale is equivalent for wild type and *clf* within each gene. **(B)** Scatter plot of log2 fold change in *clf* over wild type. y = x line represents non-differential induction of iron acquisition genes between wild type and *clf*. **(C)** Density histogram of differentially induced FIT- and PYE-dependent iron acquisition genes between wild type and *clf*.

**FIGURE 3:**
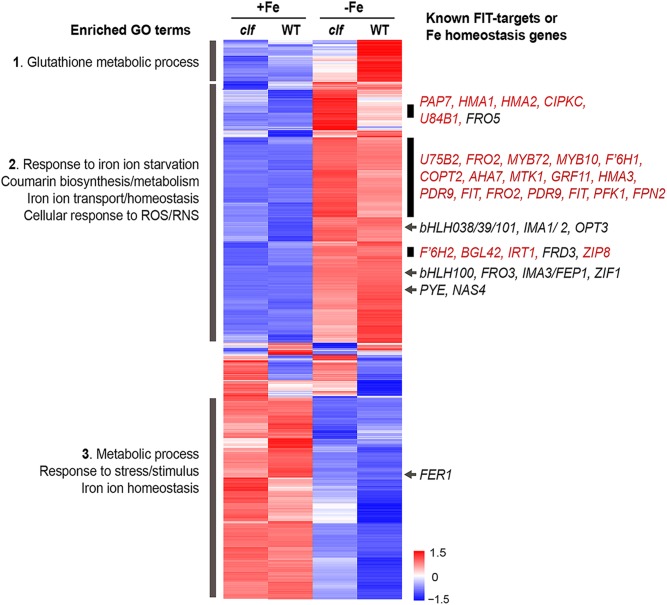
Cluster analysis of genes differentially regulated by iron in wild type and *clf* roots. Heat map of genes differentially regulated by iron in wild type or *clf* root tissue. Enriched GO terms and known iron homeostasis genes or FIT-regulated genes (red) in the subsets highlighted are indicated. Numbers on the scale bar represent standard deviation from the mean.

To get a broader sense of the impact of H3K27me3 on the expression of iron homeostasis genes, we conducted RNA-seq analysis with RNA isolated from root tissue of wild type and *clf* mutants treated under iron deficient and sufficient conditions. We identified 310 genes that were differentially up-regulated in iron deficient wild type roots and 581 genes in *clf* roots. As expected, the list of iron-regulated genes included the well-established iron homeostasis genes and other genes previously identified to be regulated by iron (Figure 2, 3 and Supplementary Table S2). Alignment of the read counts with the FIT-dependent iron deficiency response genes revealed greater induction of these genes in *clf* than in wild type in iron deficient conditions (Figure 2A), while PYE-dependent iron deficiency response genes were comparably induced between wild type and *clf* under iron deficiency (Supplementary Figure S2). Additionally, it is notable that even under iron sufficient conditions, the read counts for genes in the FIT pathway were consistently higher in *clf* compared to wild type, as opposed to PYE-dependent genes, for which the relative read counts in wild type and *clf* were variable (Supplementary Figure S3). This suggests that H3K27me3 takes part in repressing FIT-dependent iron acquisition genes under iron sufficient conditions. The overall read counts of these genes in iron-sufficient *clf* are still low; this signifies that iron-dependent upstream regulators are necessary to fully induce FIT-regulated iron acquisition genes, and explains why the small, yet significant changes were not detectable from our initial qRT-PCR analysis (Figure 1).

To visualize the differential expression of multiple FIT- and PYE-dependent iron responsive genes in *clf* compared to wild type, we plotted log2 fold change of *clf* over the log2 fold change of wild type for these genes, along with the line y = x (Figure 2B). The fold changes were calculated based on RNA-seq read counts in iron deficient conditions compared to iron sufficient conditions for both wild type and *clf*, and log2-transformation was conducted to obtain approximately normal distributions (Figure 2C). We observed a clear trend of FIT-dependent genes more often showing higher fold changes in *clf* than in wild type from the scatter plot and density plot (Figure 2B,C). Thus, we performed a randomization test to estimate the null distribution of the proportion of FIT-target genes that were more robustly expressed in *clf* than in wild type. This null distribution was then compared to the observed proportion FIT-dependent genes with a higher fold induction in clf than in wild type under iron deficiency. Based on the randomization test, FIT-target genes were more likely to have higher log fold changes in *clf* compared to PYE-dependent genes (*p* < 0.001; Figure 2B,C). Additionally, the induction of FIT-dependent transcripts in *clf* compared to wild type was generally more drastic than that of PYE-dependent transcripts (Mann-Whitney U test, *p* < 0.001). For example, under iron deficiency, expression of FIT-dependent genes such as *FIT*, *FRO2*, and *IRT1* were each approximately 3.9, 13.7, and 11.3-fold induced in wild type, whereas their transcript levels were each approximately 5.0, 40.8, and 22.4-fold induced in *clf* roots (Supplementary Table S2). On the other hand, fold change inductions of PYE-dependent genes were similar between the two genotypes, with transcript levels of *PYE*, *BTS*, and *FRO3* about 4.9, 5.9, and 17.5 times higher in wild type under iron deficiency and 4.6, 5.7, and 16.4 times higher in *clf* under iron deficiency respectively (Supplementary Table S2).

We conducted a hierarchical clustering analysis with 1021 genes that were differentially regulated by iron in wild type or *clf* roots. Genes differentially regulated in response to iron in the roots exhibited generally consistent expression patterns regardless of the genotype (Figure 3). From the heat map generated by the cluster analysis, we noted a region of genes that were more robustly repressed under iron sufficient conditions and expressed less under iron deficient conditions in *clf* than in wild type; this group was enriched with genes involved in glutathione metabolism (Figure 3, Group 1). Among the genes highly expressed in iron sufficient conditions but downregulated under iron deficiency, genes involved in metabolism, stress responses, and iron homeostasis were over-represented (Figure 3, Group 3). Within this group are *FER1* and *FER4*, which encodes ferritins that bind to iron to prevent iron-induced cytotoxicity (Ravet et al., 2009).

In addition, we identified a group of genes up-regulated under iron deficiency but down-regulated under iron sufficient conditions (Figure 3, Group 2). This group was enriched with genes iron deficiency response genes, such as those that participate in coumarin synthesis and iron uptake, as well as genes involved in responding to reactive oxygen/nitrogen species (ROS/RNS). Within this group, we noted subsets of genes that had markedly higher levels of transcripts in the *clf* mutant than in wild type (Figure 3). These subsets included a high proportion of FIT-dependent genes; about 24% of genes, i.e., 45 out of 191 genes, from this category were previously identified to be regulated by FIT (Colangelo and Guerinot, 2004; Mai et al., 2016). Meanwhile, iron deficiency inducible PYE-dependent genes were distributed throughout the upper half of the heat map. The expression of *bHLH38/39/100/101*, which encode proteins that form heterodimers with FIT, was induced under iron deficiency in both wild type and *clf* (Figure 3). However, their induction levels were not significantly different between wild type and *clf* (Figure 3). This observation is consistent with our hypothesis that PRC2 affects FIT-regulated genes. In addition, it reveals that *bHLH38/39/100/101* were present and would be able to interact with FIT in both genotypes.

Overall, the RNA-seq results corroborated our findings from the qPCR analysis, confirming that iron acquisition genes were more highly induced by iron deficiency in roots of *clf* loss-of-function mutants compared to wild type. Furthermore, the statistical analyses with fold change induction of iron deficiency response genes (Figure 2) and the clustering analysis of genes differentially regulated by iron (Figure 3) consistently revealed that FIT-dependent genes are disproportionately more highly induced by iron deficiency in *clf* roots. Such findings from the RNA-seq analysis and our qRT-PCR data (Figure 1) strongly suggest that H3K27me3 is attenuating the induction of FIT-regulated genes under iron deficiency.

### H3K27me3 Is Deposited on FIT-Dependent Iron Deficiency Response Genes

We conducted chromatin immunoprecipitation followed by qPCR (ChIP-qPCR) using antibodies against H3K27me3 and primers specific to multiple regions of the Strategy I genes, *FIT*, *FRO2*, and *IRT1*, (1) to confirm that these FIT-dependent genes are direct targets of H3K27me3, and (2) to test if H3K27me3 is differentially deposited on these gene loci depending on the iron status of the plant. Primers were designed to target upstream regions of exon 1 in order to perceive maximal H3K27me3 enrichment, which occurs primarily at the promoters and transcribed genic regions (Zhang et al., 2007). If the FIT-dependent iron deficiency response genes are direct targets of H3K27me3, H3K27me3 should be highly enriched at their loci in wild type. Substantially less H3K27me3 deposition is expected in *clf* regardless of the iron conditions. Additionally, if H3K27me3 is involved in transcriptionally regulating FIT-dependent genes in response to iron, we expected to see differential levels of H3K27me3 enrichment under iron deficient and sufficient conditions.

**FIGURE 4:**
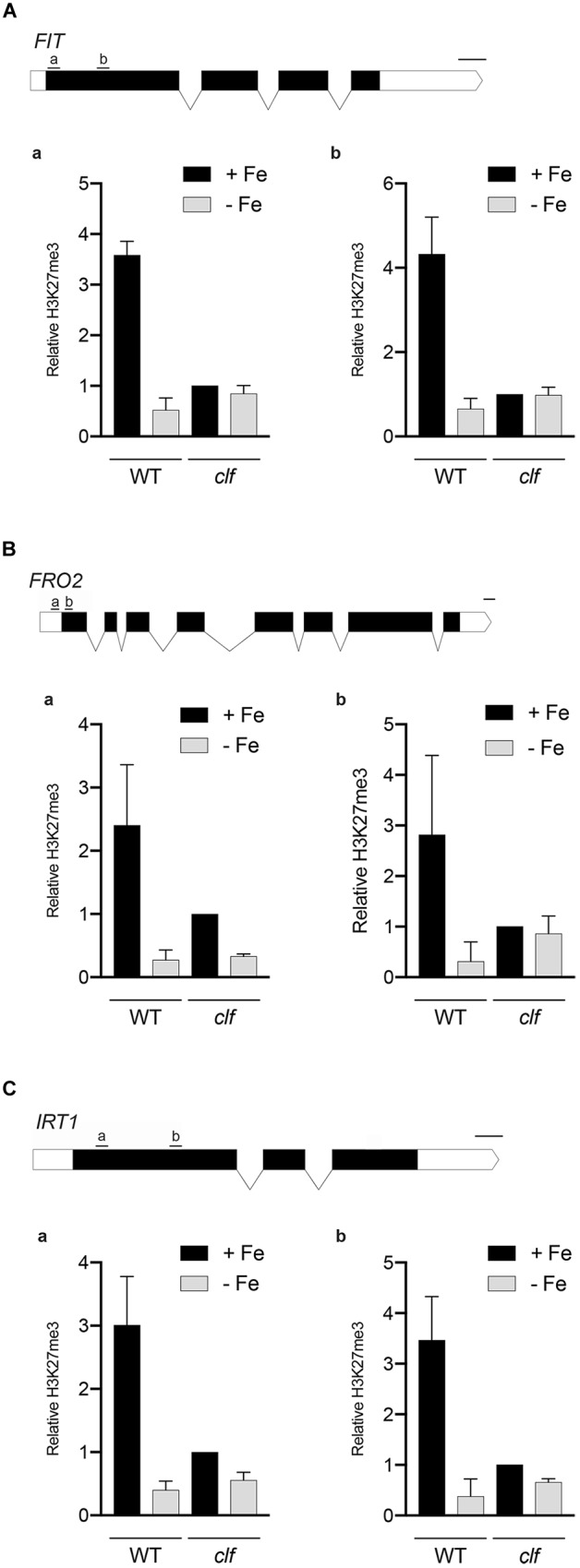
H3K27me3 enrichment at the loci of iron acquisition genes. H3K27me3 deposition at the indicated regions **(a,b)** of **(A)** *FIT*, **(B)** *FRO2*, and **(C)** *IRT1* gene loci was detected by ChIP-qPCR from wild type and *clf* roots treated under iron deficient (–Fe) or iron sufficient (+Fe) conditions. ChIP-qPCR signal was normalized with input DNA, and H3K27me3 enrichment relative to that in *clf* grown under iron-sufficient conditions was plotted. Error bars represent standard error of the mean of three biological replicates (*n* = 3). Scale bar above gene diagram represents length for 100 bp.

According to our ChIP-qPCR results, H3K27me3 enrichment at the *FIT*, *FRO2*, and *IRT1* loci was distinctly higher in wild type grown in iron sufficient conditions than in iron deficient wild type or *clf* from either iron condition (Figure 4). This provides evidence that the FIT-dependent iron deficiency response genes are direct targets of PRC2-mediated H3K27me3, consistent with the large scale epigenomics studies in which *FIT*, *FRO2*, and *IRT1* were identified as H3K27me3 targets (Table 1). Furthermore, our results suggest that H3K27me3 is involved in regulating the iron deficiency response at multiple levels, at the *FIT* locus as well as downstream of FIT at the *FRO2* and *IRT1* loci.

### *clf* Seedlings Are More Resistant to Low Iron Conditions Than Wild Type

**FIGURE 5:**
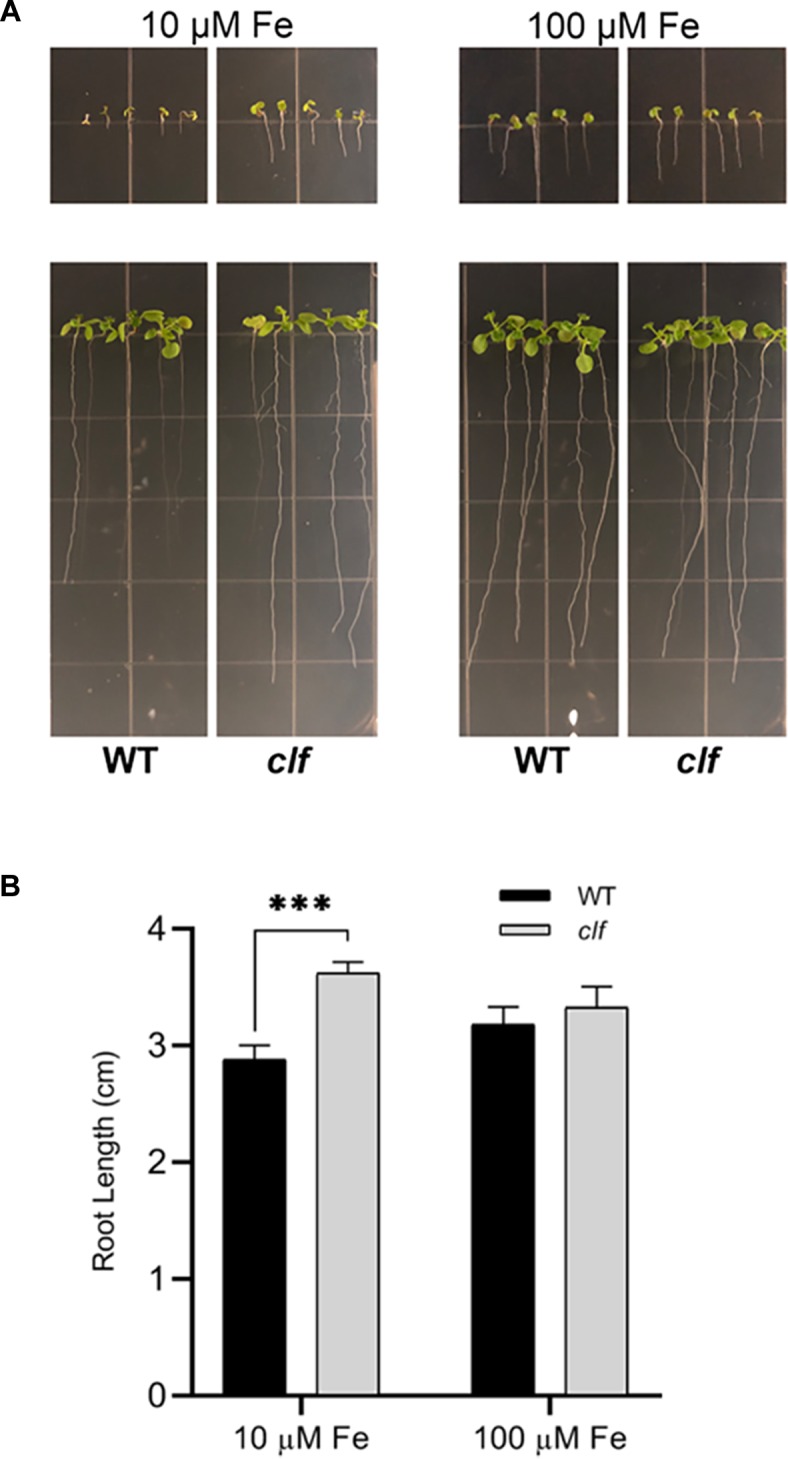
Growth phenotypes of *clf* and wild type under low iron conditions. **(A)** Wild type and *clf* plants were germinated and grown on iron-limiting (10 μM Fe) and iron-sufficient (100 μM Fe) media. Photos in the upper panel were taken 4 days after germination, and Photos in the lower panel were taken 10 days after germination. **(B)** Quantified root length. Mean values of root lengths are shown with standard error (*n* = 20–24; ^∗∗∗^*p* < 0.001).

Our results showed that FIT-dependent iron acquisition genes are more robustly induced in *clf* roots upon iron deficiency (Figure 1–3) and that they are subjected to CLF-mediated H3K27me3 (Figure 4). Therefore, we hypothesized that *clf* might be more tolerant to iron deficiency and examined the young seedlings of *clf* and wild type germinated on media that lack iron or are supplemented with a low concentration of iron that is suboptimal for growth. Consistent with our hypothesis, *clf* seedlings grew better than wild type on iron-limiting media, whereas the growth of both wild type and *clf* plants were comparable under iron sufficient conditions (Figure 5A). The root length of *clf* mutants was considerably greater than that of wild type when the plants were germinated and grown on low iron media (Figure 5B and Supplementary Figure S4), which correlated with their high level of iron acquisition gene expression (Figure 1 and Supplementary Figure S1). The overall growth and quantified root length were comparable between wild type and *clf* grown on iron sufficient media (Figure 5 and Supplementary Figure S4), suggesting that the difference in growth observed under low iron conditions was indeed an iron effect rather than a difference due to the genotype. In media without iron, the growth of both wild and *clf* plants was severely impaired (Supplementary Figure S5). Consequently, the phenotype of *clf* in low iron suggests that iron acquisition genes are not only robustly induced at the transcriptional level, but also that their enhanced gene expression contributes to better growth under low iron conditions.

## Discussion

Precise regulation of gene expression in response to specific cues is fundamental to vital biological processes. At the transcriptional level, transcription factors and chromatin structure controlled by histone modifications have profound effects on gene expression (Li et al., 2007; Farnham, 2009). The iron deficiency response in plants and subsequent iron acquisition is, to a great extent, regulated at the transcriptional level. Indeed, multiple key transcription factors involved in iron homeostasis have been extensively studied (Gao et al., 2019). However, the impact of chromatin structure on transcriptional regulation of iron homeostasis genes remains to be understood. In this study, we provide evidence that the well-established H3K27me3 repression mark contributes to transcriptional regulation of iron deficiency by fine-tuning the induction of *FIT*-target genes in iron deficient roots.

**FIGURE 6:**
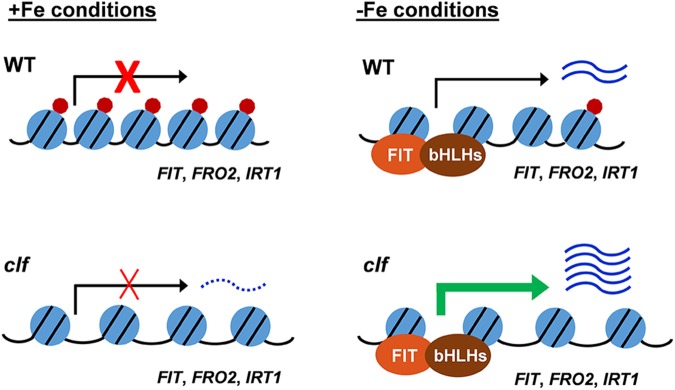
Proposed model for H3K27me3 regulation of the FIT-dependent iron deficiency response. Under iron sufficient conditions, the H3K27me3 deposition on FIT-dependent iron acquisition gene loci prevents the iron deficiency response. In response to iron deficiency, H3K27me3 is partially removed, allowing for the induction of the FIT-dependent acquisition genes. In the *clf* mutant, the H3K27me3 level is significantly reduced. Under iron sufficient conditions, the substantially reduced level of H3K27me3 marks allows for a low-level transcription of FIT-dependent genes and leads to the greater induction of iron acquisition genes under iron deficiency compared to wild type. Under iron sufficient conditions, despite the increased basal level of transcripts in *clf*, the absence of H3K27me3 is not sufficient to induce FIT-dependent iron deficiency response genes as the presence of iron negatively regulates the induction of *FIT*. Blue circles with black lines represent nucleosomes, with core histones and chromatin DNA, red circles represent H3K27me3 deposition, and the distance between blue circles depict euchromatin or heterochromatin structure due to H3K27me3. Arrows indicate active or inactive transcriptional states. Wavy lines represent transcripts of the FIT-dependent iron acquisition, with the dotted wavy line representing basal level of transcription in *clf*.

Based on our findings, we propose a working model for H3K27me3-mediated regulation of the FIT-dependent iron deficiency response (Figure 6). Under iron sufficiency, *FIT* is directly targeted by H3K27me3, resulting in repression of its transcription and the subsequent iron deficiency response. The removal of the trimethylation mark is still insufficient for the full induction of *FIT* and its downstream targets, as evidenced by the low expression of these genes in *clf* in the presence of iron (Figure 1A, 3). This implies the involvement of regulatory mechanisms upstream of *FIT* that are responsive to iron. Despite the higher level of *FIT* transcripts detected in iron sufficient *clf* compared to wild type (Supplementary Figure S2), without the iron deficiency signal that activates FIT, it is likely that neither *FIT* nor its downstream genes could be fully induced. Upon encountering iron deficiency, H3K27me3 is removed and the iron deficiency signal activates regulators of FIT, which then lead to the pronounced expression of *FIT* and its downstream targets. However, residual H3K27me3 may attenuate the induction of iron acquisition genes in wild type. In *clf*, where there is very little H3K27me3 deposition, *FIT* is more strongly induced than in wild type under iron deficient conditions. Additionally, *FRO2* and *IRT1* are also direct targets of H3K27me3. Therefore, the high-level induction of *FIT* and the lack of H3K27me3 on the *FRO2* and *IRT1* loci are likely to contribute to the augmented induction of these FIT-dependent genes in *clf* roots compared to wild type.

We hypothesize that PRC2-mediated H3K27me3 provides a mechanism to limit the maximum level of induction of iron acquisition. This would be beneficial for plants, as cellular iron levels must be maintained within a very narrow range due to iron’s redox properties that can facilitate the production of cytotoxic hydroxyl radicals (Halliwell and Gutteridge, 1992). Multiple studies have shown that the expression of iron acquisition genes is very precisely controlled depending on the iron status of the plant. *FIT*, *FRO2*, and *IRT1* are post-transcriptionally regulated to ensure that their proteins do not accumulate or stay active in the presence of iron (Connolly et al., 2002; Connolly et al., 2003; Colangelo and Guerinot, 2004; Kerkeb et al., 2008; Sivitz et al., 2011; Shin et al., 2013). Additionally, as cytotoxic effects can be caused by the entry of non-iron secondary substrates, IRT1 also undergoes monoubiquitin-dependent endocytosis (Barberon et al., 2011, 2014) and acts as a transceptor that directly senses excess non-iron metals to control self-degradation (Dubeaux et al., 2018). In addition to the regulation at the post-transcriptional and post-translational levels, our findings indicate that a mechanism to avoid overaccumulation of iron is imposed at a much earlier stage prior to transcription. Furthermore, we speculate that H3K27me3 contributes to preventing iron-induced toxicity in wild type roots under iron sufficient conditions, supported by proof of greater induction of FIT-dependent genes in *clf* compared to wild type in iron sufficient conditions (Supplementary Figure S3). We noted that H3K27me3 deposition at the loci of iron acquisition genes in wild type and *clf* under iron deficiency were comparable based on our ChIP-qPCR results (Figure 5). However, this may only appear to be the case due to the limited resolution afforded by probing specific regions by ChIP-qPCR. H3K27me3 deposition may occur at other locations along the gene, and a higher resolution assay such as ChIP-seq may reveal nuances in H3K27me3 enrichment that were not sufficiently detected by the ChIP-qPCR. As a result, it is likely that there is considerably decreased H3K27me3 enrichment on *FIT* and FIT-target genes in *clf* than in wild type under iron deficiency.

Environmentally induced chromatin remodeling has been studied in plants. The best understood example is for vernalization, in which PRC2-mediated chromatin modifications on *FLC* silences its expression in response to prolonged cold (Michaels and Amasino, 1999; Lee et al., 2015). Recently, more research has been conducted to investigate changes in chromatin structure in response to a wide range of abiotic stressors, and changes in histone modification and chromatin remodeling in response to nutrient availability have been observed (Probst and Mittelsten Scheid, 2015; Secco et al., 2017). For example, *NITRATE TRANSPORTER 2.1* (*NRT2.1*), which encodes a high-affinity root nitrate transporter, was found to be subjected to transcriptional repression by PRC2-mediated H3K27me3 (Bellegarde et al., 2018). H3K27me3 down-regulates *NRT2.1* expression under nitrogen limiting conditions, where the gene is most highly expressed.

Transcriptional regulation of a few iron homeostasis genes by histone modifications have been reported. *FERRIC REDUCTASE DEFECTIVE 3* (*FRD3*), which encodes the citrate effluxer that facilitates iron translocation in the xylem (Durrett et al., 2007), was identified as a direct target of GCN5 (Xing et al., 2015). GCN5 mediates acetylation of H3K14 and facilitates the acetylation of H3K9 and H3K27, which is involved in transcriptional activation of a large number of genes (Vlachonasios et al., 2003; Earley et al., 2007; Benhamed et al., 2008). GCN5 was proposed to regulate citrate efflux in root tissues via modulating *FRD3* expression when iron is limited (Xing et al., 2015). However, the induction of iron acquisition genes, such as *IRT1* and *FRO2*, under iron deficiency was not regulated by GCN5 (Xing et al., 2015). Expression of several Ib subgroup bHLH genes involved in the iron uptake process, such as bHLH38, bHLH39, bHLH100, and bHLH101, was reported to be negatively regulated by SKB1-mediated histone H4R3 dimethylation (Fan et al., 2014). It was also observed that *FRO2* and *IRT1* expression was higher in *skb1-1* than in wild type roots under iron deficient and sufficient conditions; nevertheless, *FRO2* and *IRT1* were not direct targets of SKB1 (Fan et al., 2014).

Our study provides a novel understanding of the transcriptional regulation of iron uptake in Arabidopsis by showing that H3K27me3 works upstream of *FIT* to partially repress the FIT-mediated iron deficiency response during iron sufficiency by directly modulating the *FIT*, *FRO2*, and *IRT1* loci. However, we still need to understand the interactions between the H3K27me3 repression mark and the iron-dependent regulatory pathway upstream of FIT. Considering that H3K27me3-mediated gene repression can be associated with tissue-specific expression of its targets (Lafos et al., 2011), further studies should address if H3K27me3 plays a role in the tissue-specific expression of FIT-dependent iron acquisition genes in the epidermis. Integrative studies to assess the combinatorial view of repression and activation marks will be necessary, and whether iron-induced changes in chromatin are heritable should also be elucidated. A better comprehension of the molecular mechanisms behind chromatin remodeling in response to iron homeostasis will provide critical insights for improving iron nutrition in plants.

## Data Availability

The RNA-seq data discussed in this publication have been deposited in NCBI’s Gene Expression Omnibus (Edgar et al., 2002) and are accessible through GEO Series accession number GSE126782 (http://www.ncbi.nlm.nih.gov/geo/query/acc.cgi?acc=GSE126782).

## Author Contributions

JJ and JL conceived the idea. JJ supervised the project. EP and KT conducted the qRT-PCR. EP performed the ChIP-qPCR and prepared the RNA samples for RNA-seq by Novogene. KT performed the low iron growth assays. FH conducted the statistical analyses. EP and JJ primarily wrote the manuscript. All authors reviewed and approved the manuscript.

## Conflict of Interest Statement

The authors declare that the research was conducted in the absence of any commercial or financial relationships that could be construed as a potential conflict of interest.

## Footnotes

1 www.arabidopsis.org
