# Supplementary material for: PRC2-Mediated H3K27me3 Contributes to Transcriptional Regulation of FIT-Dependent Iron Deficiency Response
Source: Front Plant Sci. 2019 May 16;10:627. doi: 10.3389/fpls.2019.00627 (PMC6532572; doi:10.3389/fpls.2019.00627)
Supplement: Supplementary file 2 [file Table_1.pdf]

Supplementary Table S1: Sequences of qRT-PCR primers.

Gene	Forward Primer	Reverse Primer
ACT2	5’-CCAAGCTGTTCTCTCCTTGTACGC-3’	5’-TCACCAGAATCCAGCACAATACC-3’
BTS	5’-GAAGAATTGCAAGCTGGAGCAC-3’	5’-TCCTTTGCAAGCCCAACCAGAG-3’
IRT1	5’-ACTTCAACTGCGCCGGAAGAATG-3’	5’-AGCTTTGTTGACGCACGGGTTC-3’
FIT	5’-AGCTCTCCTTCTCCGGACACATAC-3’	5’-GCTCTGTTCGAAGCATGTCCCATC-3’
FRO2	5’-GCTTCCGCCGATTTCTTAAGGC-3’	5’-AACGGAGTTATCCCGCTTCCT-3’
FRO3	5’-CCATACCTTTGTCACCATCACTCC-3’	5’-ATCCAGCCTTGCTTGCCATAAG-3’
F6’H1	5’-ACGACTGTTAGGTTTGGGACGAG-3’	5’-TCCTGCAGATATCAGGCCAGAAC-3’
NAS4	5’-ATGCTGAGAAGCGCTCATGGAC-3’	5’-CGTTCGAGAGTCACAAGGCTCAACG-3’
PYE	5’-TCTCACCGGAGAAAGAAAGGTGAC-3’	5’-GCAGTAGCATTTGCTTCATCAGAC-3’
