# Supplementary material for: PRC2-Mediated H3K27me3 Contributes to Transcriptional Regulation of FIT-Dependent Iron Deficiency Response
Source: Front Plant Sci. 2019 May 16;10:627. doi: 10.3389/fpls.2019.00627 (PMC6532572; doi:10.3389/fpls.2019.00627)
Supplement: Supplementary file 3 [file Table_3.pdf]

Supplementary Table 3: Sequences of ChIP-qPCR primers

Gene	Forward Primer	Reverse Primer
IRT1 Exon 1 (a)	5’- ACTTCAACTGCGCCGGAAGAATG -3’	5’- AGCTTTGTTGACGCACGGGTTC -3’
IRT1 Exon 1 (b)	5’- TCTTGAAGAGAACCCGTGGCA -3’	5’- CCAACTGCGTTCTTGCTGGT -3’
FRO2 5’ UTR (a)	5’- TTCTCACTAAAGCGATCGTACCG -3’	5’- TCTTCTCCTGCAGATGGGTTC -3’
FRO2 Exon 1 (b)	5’- TCGAACCCATCTGCAGGAGAA -3’	5’- CGCAGATGTGGCAACCACTTG -3’
FIT Exon 1 (a)	5’- GGAAGAGTCAACGCTCTGTCA -3’	5’- AGAACTGGGTTTTCGTCAATGGT -3’
FIT Exon 1 (b)	5’- CATCCCAACACCTGTCGATGA -3’	5’- ACCGTCGAAGCTACGGAATG -3’
