# Supplementary material for: PRC2-Mediated H3K27me3 Contributes to Transcriptional Regulation of FIT-Dependent Iron Deficiency Response
Source: Front Plant Sci. 2019 May 16;10:627. doi: 10.3389/fpls.2019.00627 (PMC6532572; doi:10.3389/fpls.2019.00627)
Supplement: Supplementary file 4 [file Image_1.pdf]

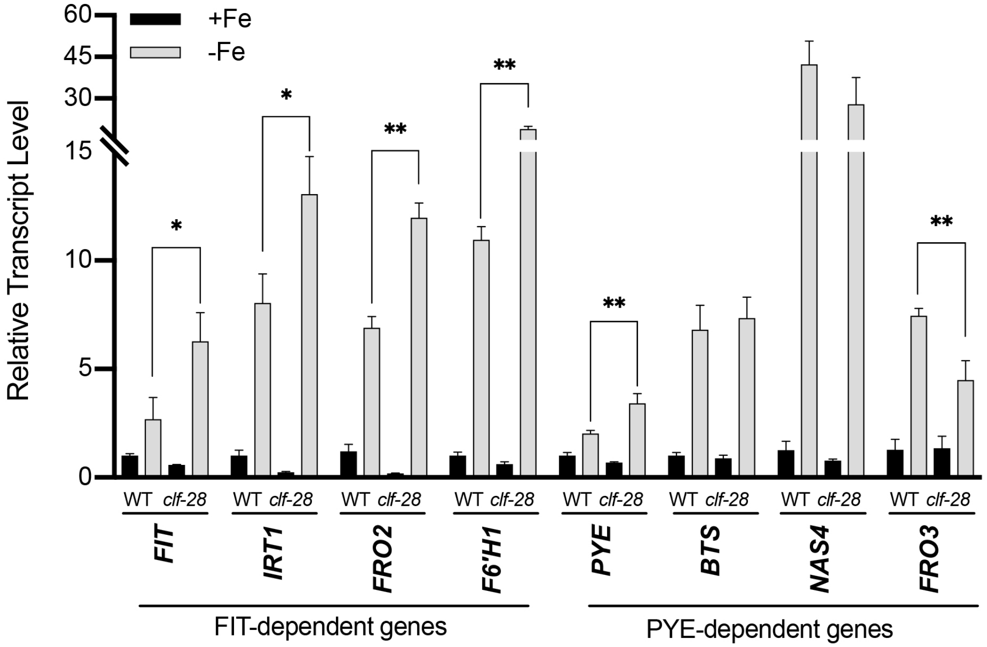

Figure S1. Transcript levels of iron deficiency inducible genes in wild type and *clf-28* (SALK_139371) roots. Quantitative RT-PCR was conducted with total RNA extracted from roots of 12-day-old plants grown on B5 without sucrose and then transferred to iron sufficient (+Fe; 100 μM Fe) or iron deficient (-Fe; 300μM ferrozine) media for 3 days. Transcript levels of FIT-dependent genes and PYE-dependent genes were normalized to an internal control, *ACT2*, and reported as the expression ratio relative to wild type under iron-sufficient conditions. Mean values of three biological replicates are shown. Error bars represent standard error. (* $p < 0.05$; ** $p < 0.01$).
