# Supplementary material for: PRC2-Mediated H3K27me3 Contributes to Transcriptional Regulation of FIT-Dependent Iron Deficiency Response
Source: Front Plant Sci. 2019 May 16;10:627. doi: 10.3389/fpls.2019.00627 (PMC6532572; doi:10.3389/fpls.2019.00627)
Supplement: Supplementary file 5 [file Image_2.pdf]

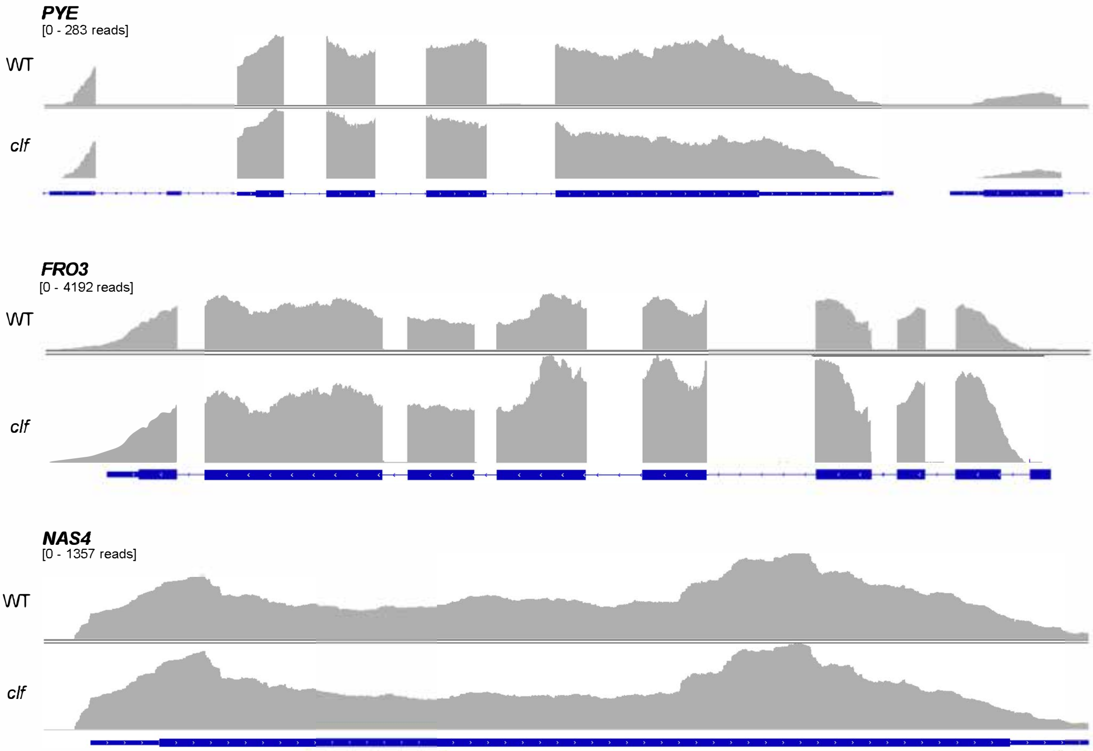

Figure S2. RNA-seq read count histograms for PYE-dependent genes under iron deficiency. Gene diagrams depict introns (line) and exons (boxes) and are aligned with read counts. The read count range, denoted below the gene name, were scaled to the maximum number of reads obtained in *clf*. Read count scale is equivalent for wild type and *clf* within each gene.
