# Supplementary material for: PRC2-Mediated H3K27me3 Contributes to Transcriptional Regulation of FIT-Dependent Iron Deficiency Response
Source: Front Plant Sci. 2019 May 16;10:627. doi: 10.3389/fpls.2019.00627 (PMC6532572; doi:10.3389/fpls.2019.00627)
Supplement: Supplementary file 7 [file Image_4.pdf]

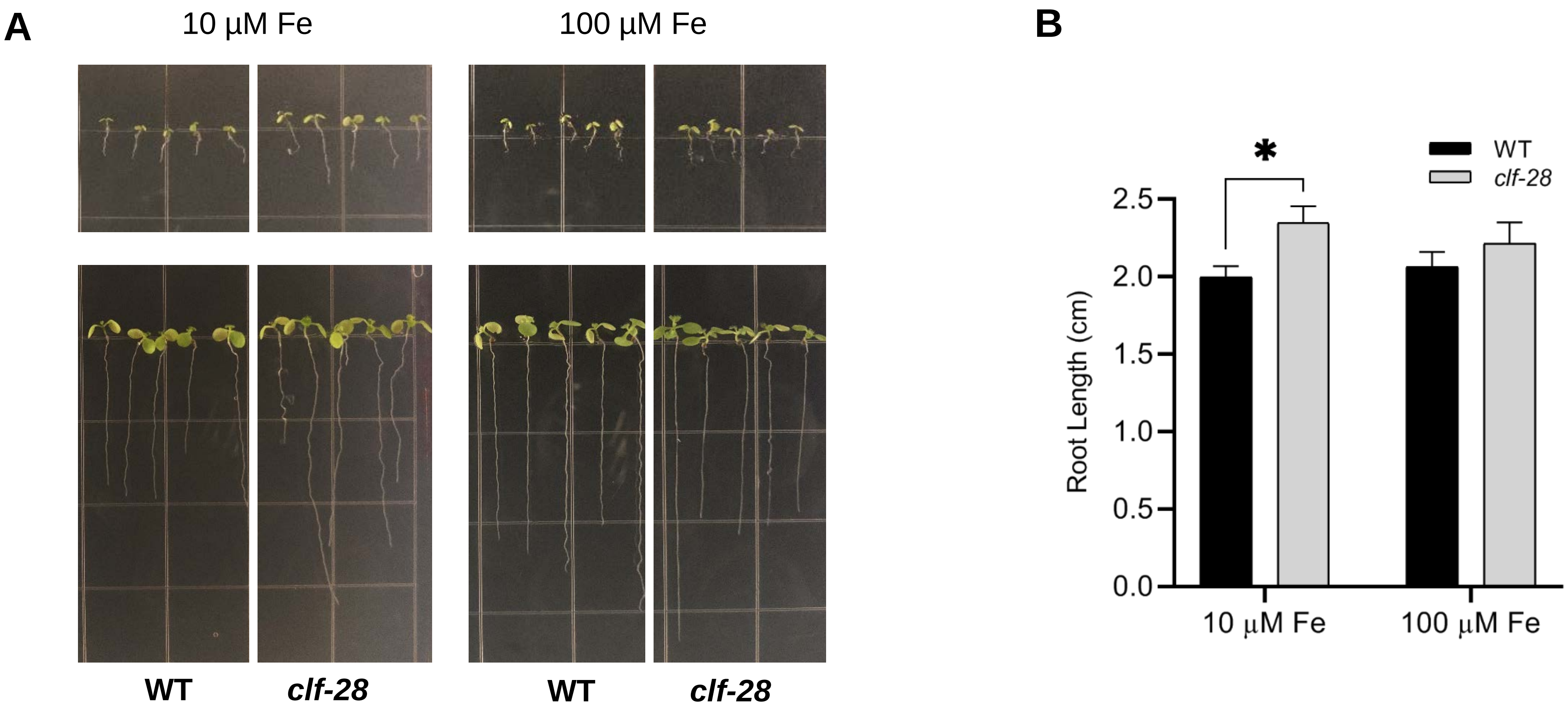

Figure S4. Growth phenotypes of a second *clf* allele, *clf-28,* and wild type under low iron conditions. **(A)** Wild type and *clf* plants were germinated and grown on iron-limiting (10 µM Fe) and iron-sufficient (100 µM Fe) media. Photos in upper panel were taken 4 days after germination, and photos in lower panel were taken 10 days after germination. **(B)** Quantified root length. Mean values of at least 29 seedlings are shown with standard error (* $p < 0.05$).
