# Supplementary material for: PRC2-Mediated H3K27me3 Contributes to Transcriptional Regulation of FIT-Dependent Iron Deficiency Response
Source: Front Plant Sci. 2019 May 16;10:627. doi: 10.3389/fpls.2019.00627 (PMC6532572; doi:10.3389/fpls.2019.00627)
Supplement: Supplementary file 8 [file Image_5.pdf]

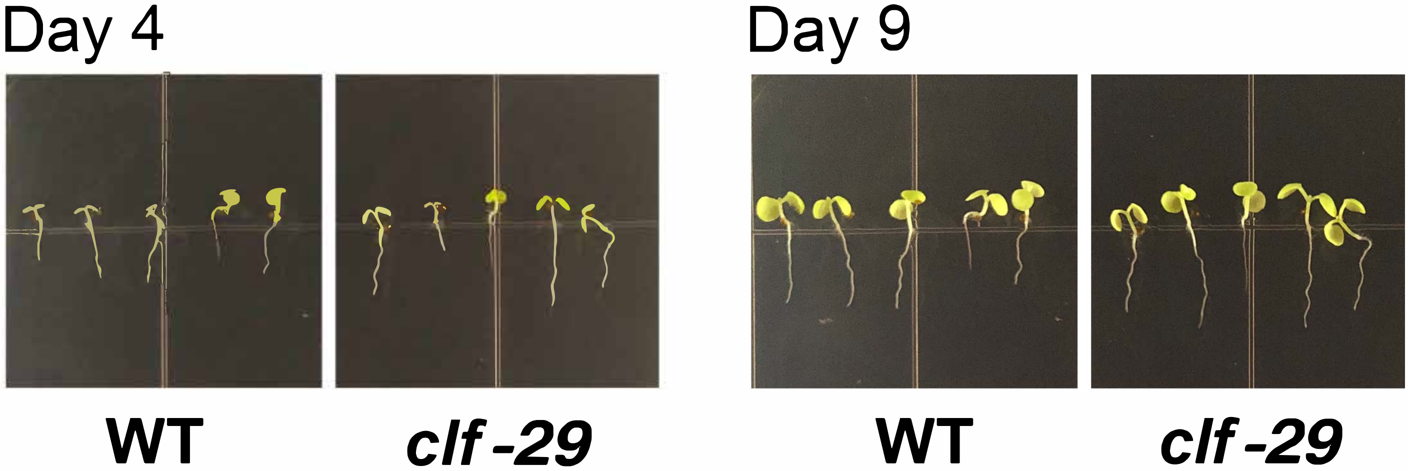

Figure S5. Wild type and *clf -29* growth in iron deficient conditions. Wild type and *clf-29* plants were germinated and grown on media with no iron (0 μM) supplemented with 300 μM ferrozine. Photos were taken 4 and 9 days after germination.
